# Commentary “Reinstatement of long-term memory following erasure of its behavioral and synaptic expression in *Aplysia*”

**DOI:** 10.3389/fpsyg.2015.01112

**Published:** 2015-08-04

**Authors:** Patrick C. Trettenbrein

**Affiliations:** ^1^Language Development & Cognitive Science Unit, Department of English Studies, University of GrazGraz, Austria; ^2^Department of Linguistics, University of GrazGraz, Austria

**Keywords:** long-term memory, learning, synaptic plasticity, Hebbian learning, connectionism, representationalism, cognitive science, neuroscience

An article published at the end of last year by Chen et al. (2014) reported their revelatory work with cultured *Aplysia* motor and sensory neurons which has led them to challenge the idea that synaptic connectivity and conductivity form the basis of learning and memory in the brain. With their paper Chen et al. have weighed in on a debate that has been raging in the cognitive sciences for most of the field's existence (Gallistel, 1998), namely the question of whether the brain should or can reasonably be considered a representational-computational system.

In a relatively recent book, Gallistel and King (2009) demonstrated how well-established findings from computer science and (computational) cognitive science might influence our thinking about computational processes in the brain. They make a very detailed and elaborate argument which cannot adequately be reproduced here without distortion. Still, in essence, Gallistel and King argue that the brain must somehow possess an architecture similar to that found in a von-Neumann machine, that is adhere to the abstract architectural properties of a universal Turing machine. They expound that an essential conceptual requirement of Turing machines, a read/write memory, has been overlooked by neuroscientists and neurobiologists in their search for the locus of memory in the brain, concluding that presently virtually nothing is actually known about how the brain encodes information, stores and retrieves information from memory, and performs computations on symbols.

Based on the work of Ramón y Cajal and Sherrington, Donald Hebb made the at first merely theoretical proposal that differences and changes in synaptic connectivity and strength might constitute the basic mechanism of how information is stored in the brain (Glickstein, 2014). The discovery of long-term potentiation (and subsequently long-term depression) provided physiological confirmation for Hebb's ideas about learning. The “key idea” behind Hebbian learning is that learning is associative in nature, meaning that learning essentially comes down to association induced by simultaneous activity of neurons or absence thereof. Large branches of the cognitive sciences have since followed this intuitive train of thought by deeming learning to be association, arguing that so-called associative long-term potentiation provides the mind/brain's mechanism for learning and memory. This has led many cognitive scientists to argue against the idea of the mind/brain as a representational-computational system (e.g., Rumelhart et al., 1986).

Admittedly, Hebbian learning has an intuitive appeal, but Gallistel and King (2009) as well as Gallistel and Matzel (2013) have convincingly argued that we run into severe problems when making synaptic conductance the locus of memory. Pavlov already discovered that a multitude of experiential aspects in combination determine the associative strength between two stimuli. All different variables are mixed and encoded in a single association. However, it is impossible to even approximately determine the value of any of the variables that entered into the original calculation, so that association is, in mathematical terms, a many-one function. Given this many-to-one nature of associative strengths, the only hope to regain useful information from them is by doing away with Pavlov's intent to discover general “laws of association” and assuming that there are different mapping rules (i.e. neurobiological processes) for *every* synapse. Clearly, this is an unpleasant assumption that no researcher wants to make. Furthermore, synaptic conductance is not accessible to the computational processes assumed to be carried out in neurons, supposedly addition of multiplicatively weighted input signals (Kandel et al., 2013). We thus see a vast discrepancy between what associative long-term potentiation is assumed to be capable of doing and what it actually can do.

From the perspective of computational cognitive science it follows that when looking for a mechanism that implements a read/write memory in the nervous system, turning to synaptic strength and connectivity patterns might be misleading. What is more, synapses might already be too complex in terms of implementing such an elementary function. As Gallistel and King put it:

In the final analysis, however, our skepticism rests most strongly on the fact that the synapse is a circuit-level structure, a structure that it takes two different neurons and a great many molecules to realize. It seems to us likely for a variety of reasons that the elementary unit in the memory mechanism will prove to be a molecular or sub-molecular structural unit. (2009, p. 282)

Based on the observation that every neuron actually performs a rather stereotypical computational operation on its input (Kandel et al., 2013), it seems likely that much more with respect to memory is going on in the cell body (see Johansson et al., 2014). Chen et al.'s (2014) work therefore provides some further tentative confirmation for this “hunch” of Gallistel and collaborators (Gallistel and King, 2009; Gallistel and Matzel, 2013; Gallistel and Balsam, 2014).

All in all, it seems that there indeed are two different processes at work in learning and memory, as Chen et al. (2014) also point out. While the exact details about both remain obscure, there appears to be a dissociation between the way in which learning occurs and how memory works. We do not know how the brain implements a read/write memory, but there is good evidence that it does. Similarly, there is ample and convincing evidence, also in Chen et al. (2014), that synaptic conductivity and connectivity play a role in regulating behavior. Consequently, it appears that synaptic plasticity might not so much be a precondition for learning as it is a consequence of it, so that the observed rewiring of synaptic connections might constitute the brain's way of ensuring an “efficient,” or possibly even close to “optimal” (Cherniak et al., 2004; Sporns, 2012), connectivity and therefrom resulting activity pattern that is appropriate to environmental (and presumably also “internal”) conditions. Synaptic plasticity thus might be reinterpreted as a way of regulating behavior (i.e., activity and connectivity patterns) only after learning has already occurred (i.e., after relevant information has been extracted from the environment and stored in memory).

Extrapolating Chen et al.'s (2014) findings stemming from work on *Aplysia* to claims about much more complex nervous systems is, of course, speculative in nature, to say the least. However, it seems to be no more speculative than the almost universally accepted idea of the synapse being the locus of memory. Similarly to Johansson et al. (2014), the work of Chen et al. (2014) shows that (1) there is plenty of “room” for the implementation of symbols other than synapses, and (2) substantiates the understanding that the network approach of connectionism might indeed best be seen as an implementational theory (Fodor and Pylyshyn, 1988) that still requires representation, computation, and a Turing architecture (i.e., a read/write memory). Gallistel and Balsam (2014) proclaimed that is was about time to rethink the neural mechanisms of learning and memory, Chen et al.'s experimental results add to the urgency of this claim.

## Conflict of interest statement

The author declares that the research was conducted in the absence of any commercial or financial relationships that could be construed as a potential conflict of interest.
